# Comparative data on emotional (psychotic) aggressive biting behavior in mice of ddY strain measured by using two devices; Aggressive response meter and powerlab-compatible type aggressive response meter

**DOI:** 10.1016/j.dib.2023.109231

**Published:** 2023-05-12

**Authors:** Kento Igarashi, Satoshi Kuchiiwa, Toshiko Kuchiiwa, Kazuo Tomita, Tomoaki Sato

**Affiliations:** a Department of Applied Pharmacology, Graduate School of Medical and Dental Sciences, Kagoshima University, 8-35-1 Sakuragaoka, Kagoshima 890-8544, Japan; b Department of Morphological Science, Field of Neurology, Graduate School of Medical and Dental Sciences, Kagoshima University, 8-35-1 Sakuragaoka, Kagoshima 890-8544, Japan; c Department of Clinical Psychology, Graduate School of Human Science, Kagoshima Immaculate Herat University, 2365 Amatatsu-Cho, Satsuma-Sendai, Kagoshima 895-0011, Japan

**Keywords:** Emotional aggression, Aggressive biting behavior, Aggressive response meter, pARM

## Abstract

The Aggressive Response Meter (ARM) has been validated for measuring emotional (psychotic) aggression triggered by mental irritation in mice. In the present article, we newly developed a device, pARM (PowerLab-compatible type ARM). We collected on the aggressive biting behavior (ABB) intensity and ABB frequency of 20 male and female mice of ddY strain studied over a period of 6 days by using pARM and the former ARM. We calculated Pearson's correlation between the values of pARM and those of ARM. The accumulated data can be referred as a basis for demonstrating the consistence of pARM and the former ARM, and used in future research to augment the understanding of stress-induced emotional aggression in mice.

**Specifications Table**

**Table:** 

Subject	Neuroscience: Behavioral
Specific subject area	Ethology, Behavioral pharmacology
Type of data	Graph Figure
How the data were acquired	The aggressive biting behavior (ABB) of isolation-reared male and female ddY mice were measured by using the Aggressive Response Meter (ARM; Muromachi kikai, Tokyo, Japan) and the newly developed device (pARM; under cooperation with Mr. Murakami, one of the ARM patent holders). The pARM is connected to the data-collecting PC through PowerLab (ADInstruments, Sydney, Australia), while the ARM is directly connected to the PC. Raw data was reposited in Mendeley data. Raw data was used to show daily transition of the values (Fig. 3) and processed to compare the values between the two devices (Fig. 4).
Data format	Raw Analyzed
Description of data collection	Data for all mice and all days used in the experiment were evaluated: there were no missing or excluded data. A comprehensive dataset was deposited Mendeley repository data.
Data source location	• Institution: Kagoshima University • City/Town/Region: Kagoshima City/Kagoshima • Country: Japan • Latitude and longitude (and GPS coordinates, if possible) for collected samples/data: 31.54748° N and 130.52503° E
Data accessibility	Repository name: Mendeley Data Data identification number: 10.17632/crhpfz9cgt.2 Direct URL to data: https://data.mendeley.com/datasets/crhpfz9cgt

**Value of the Data**

• The Aggressive Response Meter (ARM) has been validated for measuring emotional (psychotic) aggression triggered by mental irritation in mice. It is used for revealing aggressive behavior of stressed animals and for investigating the effect of psychiatric drugs on aggression.
• We collected the data on the measurement of the aggressive behavior in mice by using the newly developed PowerLab compatible-type ARM (pARM) were relevant to those of the prior device (ARM), demonstrating the consistency of both devices.
• Daily measurement of aggressive biting behavior for six days can further be utilized as a basic data for understanding the aggressive nature of isolation-reared mice.

## 1 Objective

The Aggressive Response Meter (ARM) was originally invented to examine aggressive biting behavior (ABB) of mice on inanimate objects [1,2]. It has been previously reported that 8–10 weeks of social isolation triggers elevated ABB levels in isolation-reared mice [1,2]. Additionally, stress associated with maternal separation induces elevated ABB in offspring [3]. Measurements based on the ARM have been cited as evidence of emotional aggression caused by mental irritation in both male and female mice [4,5]. The ARM device is also useful for the pharmacological analysis of drugs that reduce aggressive biting behavior [6,7] and for the analysis of aggression in histamine *N*-methyl transferase-deficient mice [8].

Recently, we developed a new device that can be connected to a PowerLab (ADInstruments, Sydney, Australia), since the ARM was discontinued in the previous manufacturer. PowerLab is an A/D converter that is widely used in laboratories around the world for data processing of instrumentation. That system inputs analog signals form sensors and amplifiers, etc., and records, analyzes, and saves the data in a computer. In the present article, we designated it pARM (PowerLab-compatible type ARM), examined ABB using the ARM and the pARM, and investigated the validity of the pARM with the conventional ARM.

## 2 Data Description

**Fig. 1:**
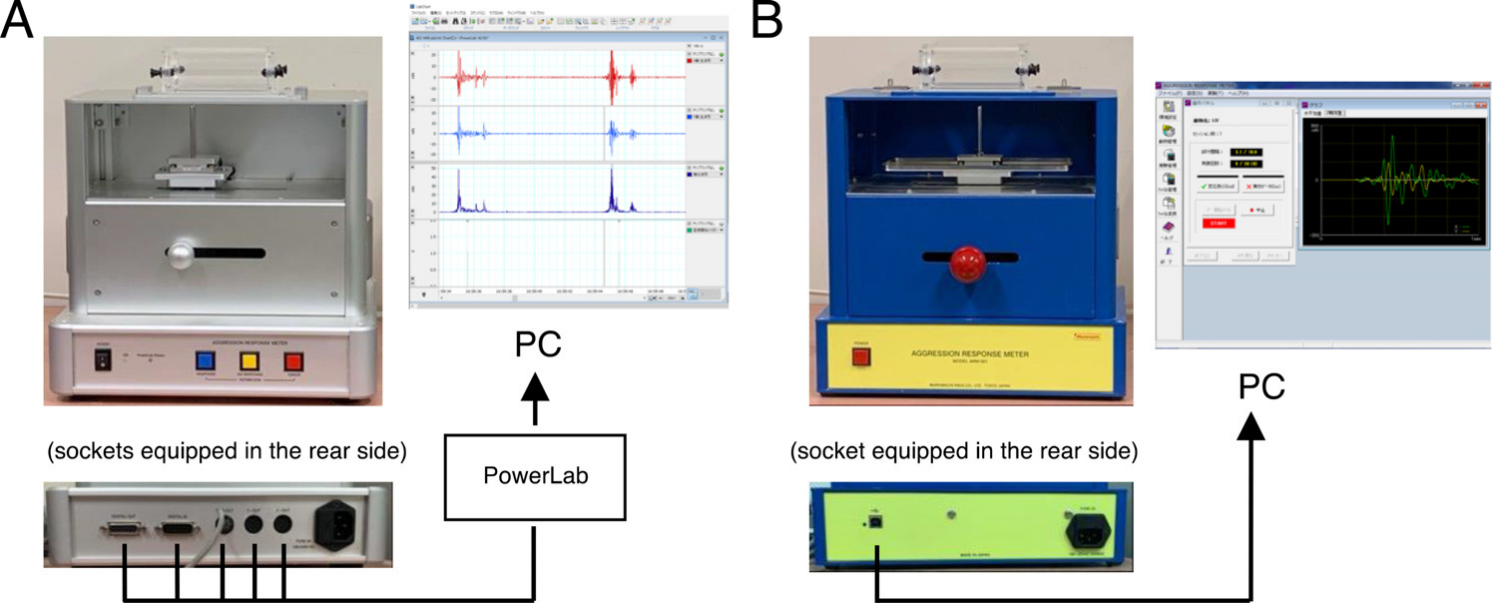
Schematic diagram showing the connection of the pARM (A) and the ARM (B). Both devices measure aggressive biting behavior (ABB) of mice. (A) The PowerLab compatible-type ARM (pARM) is connected to PC via PowerLab. (B) The ARM is internally equipped with A/D converter and is directly connected to PC.

**Fig. 2:**
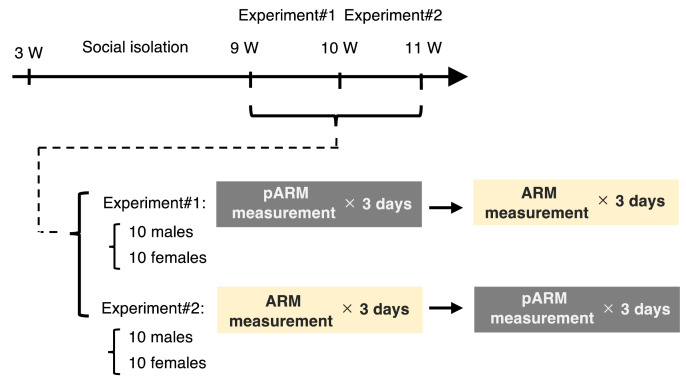
Schematic drawing of the experimental timeline in this article.

**Fig. 3:**
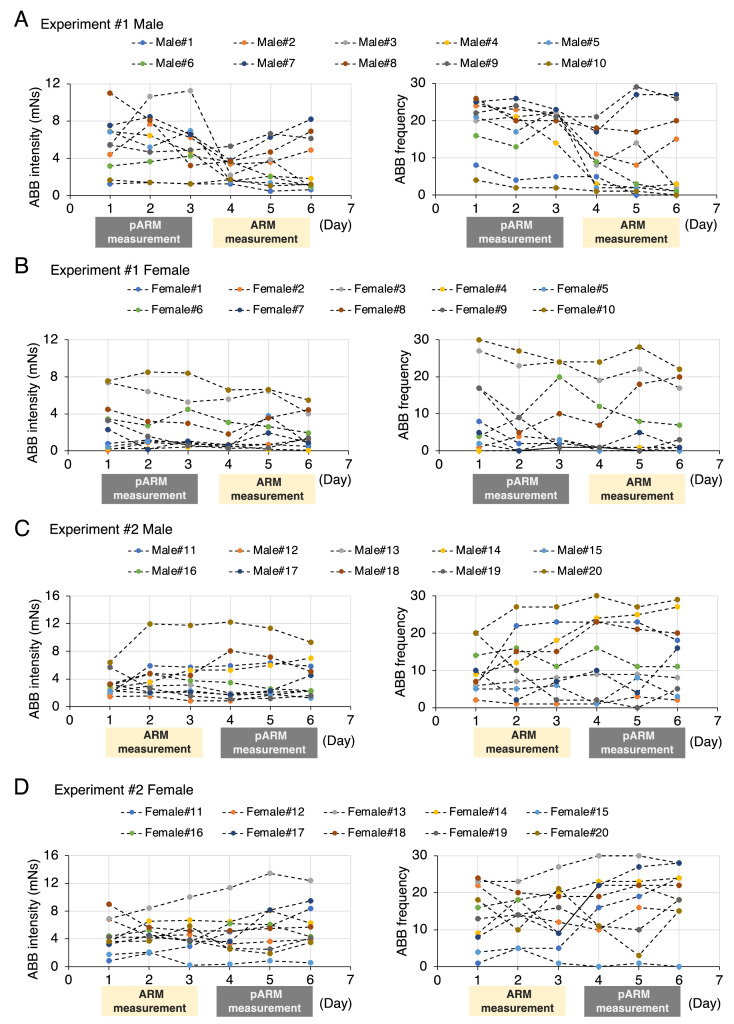
Transition of the ABB intensity and frequency of the isolation-reared mice (10 males and 10 females in each experiment: 40 animals were used in total). The values of individual animals are plotted.

**Fig. 4:**
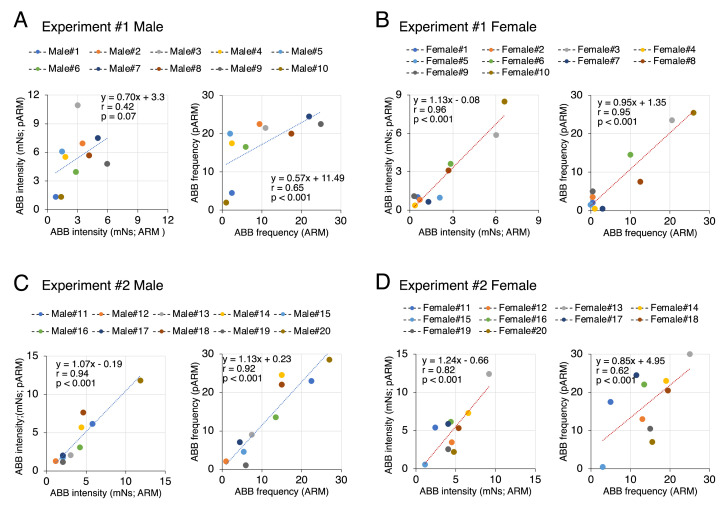
Positive correlation between ABB measured by the ARM and pARM. The dots were plotted by the averages (i.e., the average of the values in day 2 and day 3 measured by the first device and that of the values in day 4 and day 5 by the second device). Line function showing regression line, co-efficient ‘r’, and p-value are inset in each graph.

The dataset in this article describes the ABB intensity and frequency that were measured by using two devices (the ARM and the pARM) (Fig. 1). Fig. 2 describes experimental timeline in this article; we examined the ABB intensity and frequency of the socially isolated male and female mice over six consecutive days. In the experiment#1, we examined the ABB intensity and frequency of the set of 10 males (identified as male#1-male#10) and 10 females (identified as female#1-female#10) at 9 weeks in the order of the pARM and the ARM. In the experiment#2, we examined the ABB intensity and frequency of the other set of 10 males (identified as male#11-male#20) and 10 females (identified as female#11-female#20) at 10 weeks in the order of the ARM and the pARM. The collected values of the individual animals can be identified by the above-mentioned number on the Mendeley Data [9]. Fig. 3 describes the transition of the ABB intensity and frequency in each social isolation-reared mice within 6-consecutive days, which showed that isolation-reared mice exhibit unstable transition in ABB (especially in the group of Experiment#1 males). The average value of the individual animal measured by the pARM or the ARM is plotted in Fig. 4. Since Fig. 3 showed unstable nature of isolation-reared mice, we adopted the average of the values measures within the two days (i.e., the values are plotted in regard to the average of the values in day 2 and day 3 measured by the first device and that of the values in day 4 and day 5 measured by the second device). Statistically significant positive correlation was observed between the values measured by using the pARM and the ARM, for male mice group in Experiment#2, female mice group Experiment#1 and #2.

## 3 Experimental Design, Materials and Methods

### 3.1 Animals

All experiments were performed using mice of ddY strain purchased from Japan SLC (Slc: ddY; Japan SLC Inc., Shizuoka, Japan). Normal male and female mice were housed together for mating to obtain offspring. For the isolation-rearing experiments, pups were separated from the dam at 3 weeks of age and then isolated in individual cages (16 × 23 × 13 cm) until 11 weeks after birth. All animals were housed under controlled conditions (temperature:22 °C ± 2 °C; lighting: lights on at 7:00, lights off at 19:00), with food and water administered *ad libitum*. Cage exchange was performed every 10 days.

### 3.2 Measurement of ABB Intensity

The intensity and frequency of the ABB toward an inanimate object were measured using the ARM (Muromachi Kikai Co. Ltd., Tokyo, Japan), as previously described [1,^2^,7] and the pARM (Fig. 1). The pARM, that we developed under the cooperation of Mr. Murakami, one of the ARM patent holders (Tokyo Metropolitan Institute of Medical Science), is equipped with the same load sensor and can be connected to PowerLab® (26T, ADInstruments, Sydney, Australia). In the measurement using pARM, it is connected to PC via PowerLab, and the LabChart software in the PC analyzes signal from the pARM, and measures ABB intensity. The pARM is also equipped with 3 buttons in the front side, which are “Response”, “No response”, and “Error”. The usage of these buttons by experimenter is introduced in Supplementary Video. The ABB frequency (the number of ABB in the 30 trials within a session) is recorded as pushing “Response” by the experimenter.

### 3.3 Statistical analysis

Pearson's correlation co-efficient ‘r’ was employed to analyze the significance of relationship between the values of ABB examined with the ARM and the pARM. Statistical significance was set at *p* < 0.05.

## Ethics Statements

All animal procedures were approved by the Committee of Animal Experimentation, Kagoshima University (approval number: D22024). Those experiments complied with the ARRIVE guidelines and were carried out in accordance with the National Institutes of Health guide for the care and use of laboratory animals (NIH Publications No. 8023, revised 1978).

## Funding

This study was supported in part by the JSPS KAKENHI [grant number 19K19170]. The funding sources were not involved in data collection, analysis, or interpretation.

## CRediT authorship contribution statement

**Kento Igarashi:** Investigation, Data curation. **Satoshi Kuchiiwa:** Writing – original draft. **Toshiko Kuchiiwa:** Data curation, Methodology. **Kazuo Tomita:** Writing – review & editing. **Tomoaki Sato:** Writing – review & editing.

## Declaration of Competing Interest

The authors declare that they have no known competing financial interests or personal relationships that could have appeared to influence the work reported in this paper.

## Data Availability

Comparative data on aggressive biting behavior in mice of ddY strain measured by two independent devices (Original data) (Mendeley Data).

## Appendix Supplementary materials

Image, video 1
